# The impact and management of internet-based public opinion dissemination during emergencies: A case study of Baidu News during the first wave of coronavirus disease 2019 (COVID-19)

**DOI:** 10.1371/journal.pone.0299374

**Published:** 2024-04-04

**Authors:** Xin Su, Shengwen Wang

**Affiliations:** School of Business Administration, Shandong University of Finance and Economics, Jinan, Shandong Province, China; Czestochowa University of Technology: Politechnika Czestochowska, POLAND

## Abstract

**Background and aims:**

The coronavirus disease 2019 (COVID-19) public health emergency has had a huge impact worldwide. We analyzed news headlines and keywords from the initial period of COVID-19, and explored the dissemination timeline of news related to the epidemic, and the impact of Internet-based media on the public using lifecycle theory and agenda-setting theory. We aimed to explore the impact of Baidu news headlines on public attention during the first wave of COVID-19, as well as the management mechanism of regulatory departments for social public opinion.

**Methods:**

We searched Baidu News using the keywords “Novel Coronavirus” and “COVID-19” from 8 January to 21 February 2020, a total of 45 days, and used Python V3.6 to extract news samples during the first wave of the epidemic. We used text analysis software to structurally process captured news topics and content summaries, applied VOSviewer V6.19 and Ucinet V6.0 to examine key aspects of the data.

**Results:**

We analyzed the impact of Baidu News headlines on social opinion during the first wave of COVID-19 in the budding, spread, and outbreak stage of the information lifecycle. From clustering visualization and social network analysis perspectives, we explored the characteristics of Baidu News during the initial stage of the COVID-19. The results indicated that agenda-setting coverage through online media helped to mitigate the negative impact of COVID-19. The findings revealed that news reporting generated a high level of public attention toward a specific emergency event.

**Conclusions:**

The public requires accurate and objective information on the progress of COVID-19 through Baidu News headlines to inform their planning for the epidemic. Meanwhile, government can enhance the management mechanism of news dissemination, correct false and inaccurate news, and guide public opinion in a positive direction. In addition, timely official announcements on the progress of the COVID-19 outbreak and responses to matters of public concern can help calm tensions and maintain social stability.

## 1. Introduction

Public health emergencies are characterized by the sudden occurrence of an event that can potentially cause or has already caused harm to people’s physical and mental health, and which threatens social stability and development [1]. As a major public health emergency, the coronavirus disease 2019 (COVID-19) epidemic not only affected the health of the population, but also exerted a negative impact on the normal functioning of society [2]. To date, the COVID-19 epidemic has continued for 3 years, with small outbreaks still occurring from time to time. During this period, countries around the world have taken preventive and control measures to combat COVID-19, such as wearing masks, nucleic acid testing, vaccination, and quarantine protocols [3]. These measures have achieved remarkable results in containing the spread of the epidemic. However, COVID-19 has also significantly impacted the daily life and mental health of the population [4].

In early 2020, the progression of the COVID-19 epidemic quickly received substantial attention in social media and public opinion headlines [5]. If Internet-based news media had reported the outbreak in a biased manner at this time, it could have led to blind optimism or extreme pessimism among members of the public [6]. This highlights the importance of the impact and management mechanisms of Internet-based public opinion in preventing disease outbreaks and maintaining social stability [7]. In response to the spread of the COVID-19 epidemic, the Chinese government took rapid and effective measures to prevent and control the spread of the disease, to release relevant news and information in a timely manner, and to actively guide public opinion and calm people’s emotions [8].

In previous studies, researchers have analyzed data related to the epidemic published on social media platforms such as Twitter, Facebook, Weibo, and WeChat [9]. However, we identified the following gaps in existing research: (1) Few studies have used data from news search engines to explore the impact of COVID-19 on society [10]. (2) There has been little research on the mechanisms of the impact of the first wave of COVID-19-related news on public opinion and public sentiment [11]. (3) In the initial stages of public health emergencies like COVID-19, there is a requirement for a thorough analysis of the management mechanism for news reporting and dissemination on various media platforms [12]. To fill these gaps, the current study aimed to explore the public impact of Internet-based news media and its role in guiding public opinion by mining COVID-19-related headlines in Baidu News searches. Because Baidu is a Chinese-language search engine, after crawling the Chinese headlines, we systematically translated the sample data into English before analyzing its social network structure and applying time series clustering [13].

With the continuous development of Internet and media convergence technology, the manner in which news headlines are released, searched, and accessed has changed dramatically [14]. As the world’s best known Chinese search engine, Baidu responds to billions of search requests every day, and is the primary interface for many Chinese Internet users to access information and services. On the basis of Baidu’s search engine technology, Baidu News has a large user base and a wide range of searches, and has become the most powerful Chinese news search site [15]. Almost all key information released by Chinese media regarding COVID-19 can be searched in Baidu News, providing a major source of information access for the public and an important tool for researching news and public opinion [16]. Overall, Baidu News has been able to cover most headlines regarding the COVID-19 epidemic at key points in time; hence, we utilized data from Baidu News as our study sample.

Using this approach, we used data from Baidu News during the outbreak of the first wave of COVID-19 to investigate the following questions: (i) During the first wave of COVID-19, what cyclic changes occurred in the focus and reporting of Internet news regarding the epidemic? (ii) How do Internet news reports on COVID-19 impact public opinion and public sentiment? (iii) Does timely guidance and management of news-related public opinion during public health emergencies contribute to calming public sentiment and maintaining social stability? The answers to these questions may be helpful for minimizing the negative impact of COVID-19, and could serve as a valuable reference for public opinion management in future public health emergencies. To elucidate these issues, we searched COVID-19-related news headlines from Baidu News and used data measurement tools to conduct data visualization and social network analysis. We aimed to assess the development of trends during the course of the COVID-19 epidemic using agenda-setting theory and information lifecycle theory to assess the changes in Baidu News headlines during the first wave of COVID-19 in China. The ultimate purpose of the current study was to inform approaches for maintaining mental well-being by generating positive public opinion while minimizing negative emotional responses in society.

To achieve this goal, the following steps were carried out in the current study: (1) news headlines from the first wave of COVID-19 were searched and crawled in Baidu News, and the keywords, release time, and name of the media source in the sample were recorded and analyzed; (2) sample data were analyzed using topic word clustering, to which a temporal factor was added, and a visual network map of COVID-19-related news keywords was created; and (3) social network analysis was conducted on the COVID-19-related news samples, a co-word matrix was constructed, and social network centrality and parameters related to structural holes of the data were measured. The three main contributions of this study are as follows.

- Using crawler technology, this study obtained news headlines related to the first wave of the COVID-19 epidemic from Baidu News, carried out word separation processing, and conducted quantitative statistical analysis of news features, such as keywords and the reporting media outlet in the sample; we constructed a co-word matrix, and carried out social network analysis and temporal visualization on the basis of topic word clustering.
- On the basis of information lifecycle theory, we identified the different characteristics of Baidu News headlines regarding COVID-19 in the budding, spread, and outbreak stages, as well as their impact on society, and described the process of the outbreak of public opinion during the epidemic; the results revealed that the mainstream media, as opinion leaders, helped to guide the positive development of online public opinion.
- On the basis of agenda-setting theory, the results indicated that, in the face of an unexpected epidemic, mainstream Internet-based media was able to provide timely and accurate reports on the latest progress of the epidemic; at the same time, the mainstream media were able to release relevant news via the Internet to respond to the epidemic and provide timely explanations to address the concerns of the public.

**Fig 1:**
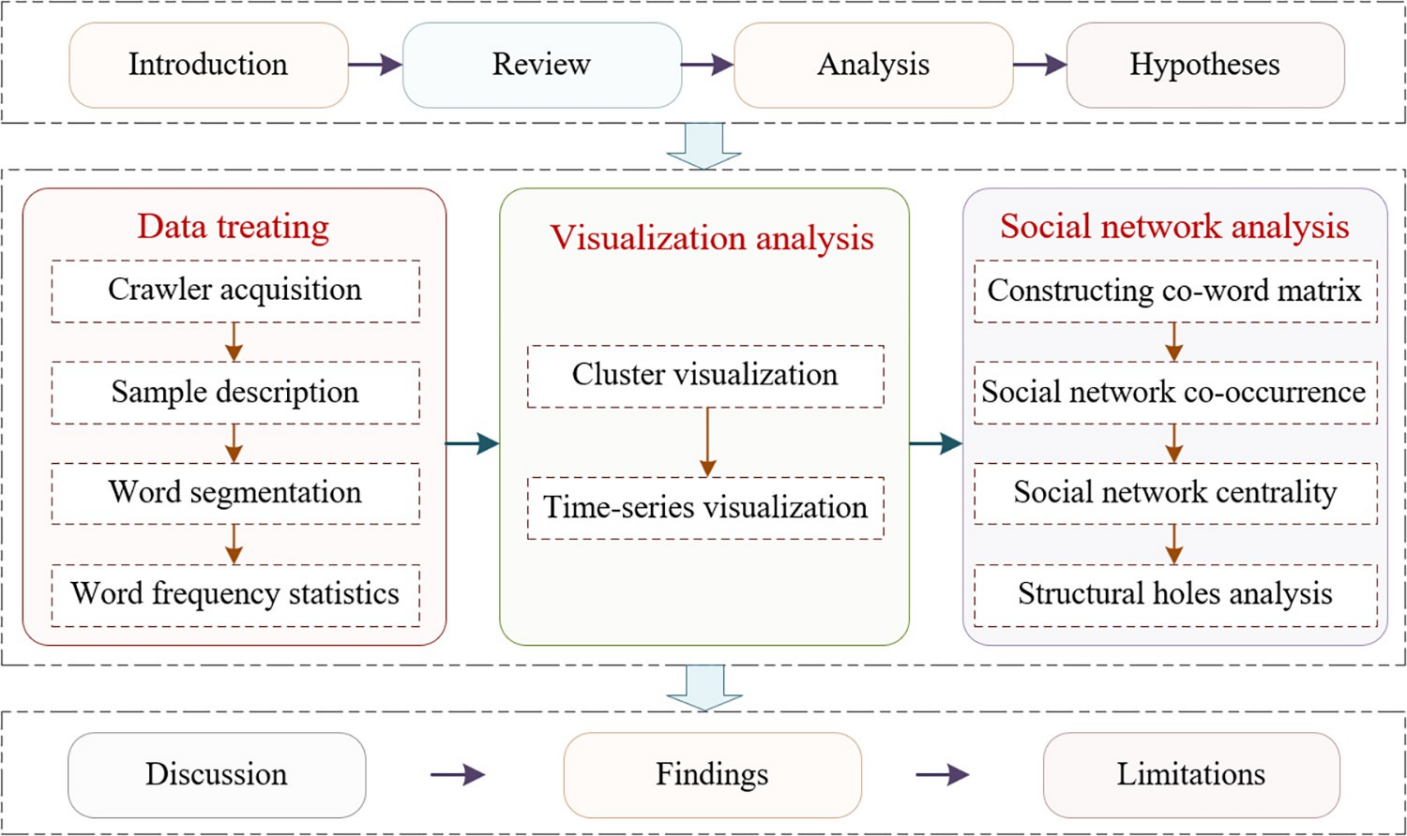
The analytical framework of this study.

The remainder of this paper is organized as follows. Section 2 reviews research on the lifecycle theory and agenda-setting theory in the context of COVID-19-related news coverage. Section 3 discusses the impact and management mechanisms underlying the dissemination of COVID-19-related public opinion. Section 4 presents the data and methods of the current study, including the data sources, sample description, word frequency statistics after word segmentation, and research methods. Section 5 presents the analysis results of the visualization of cluster and time series data, as well as social networks and structural holes. Section 6 and Section 7, respectively, provide a discussion of the research and present the conclusions that can be drawn from our findings. Fig 1 shows the analytical framework of this study.

## 2. Research review

### 2.1 Information lifecycle theory

**Fig 2:**
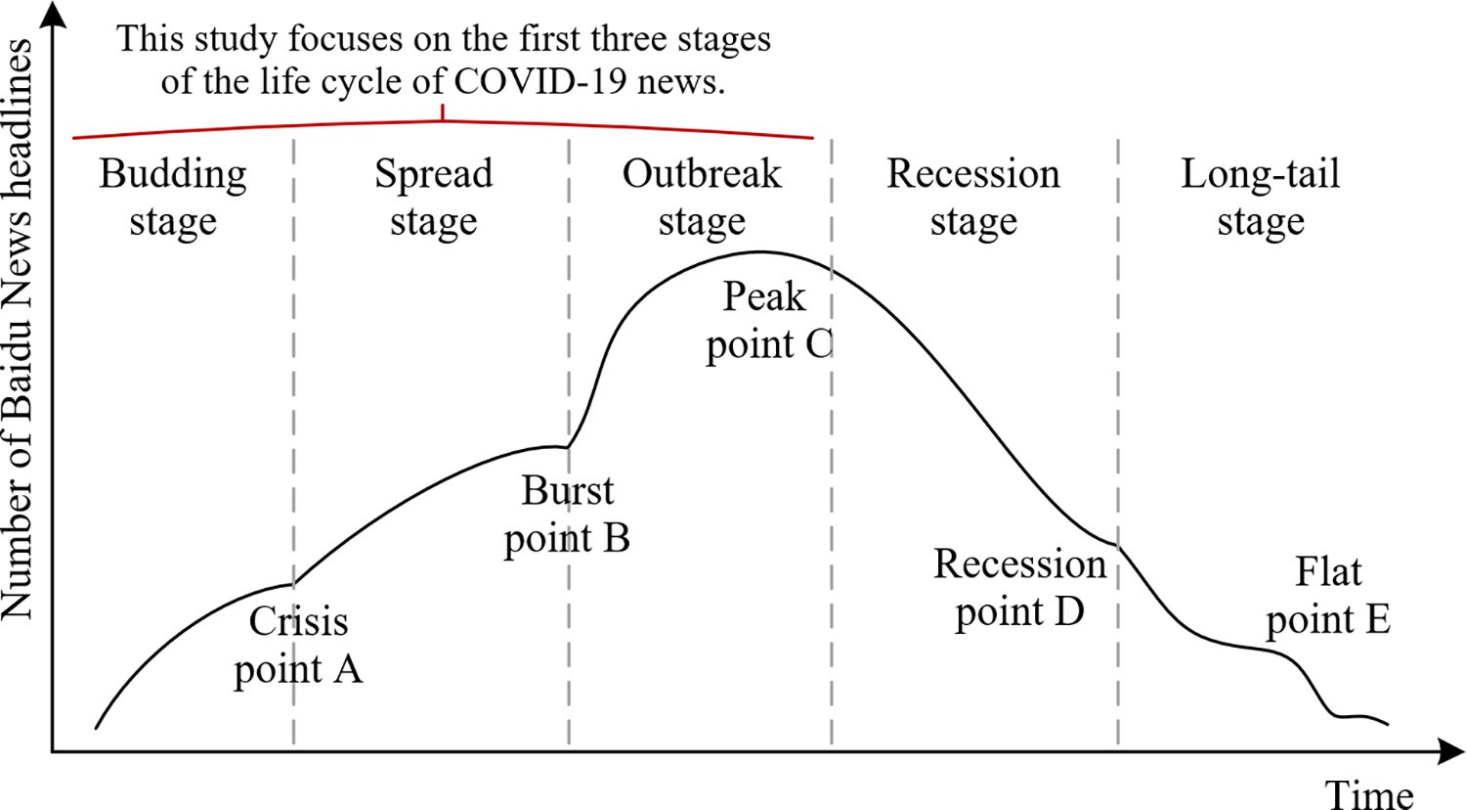
Five-stage model of the information lifecycle.

Lifecycle theory was initially applied to product iteration and economic development. In recent years, some scholars have introduced this theory to the field of emergency information management and control of public opinion [17]. The pattern of news-based public opinion fluctuation conforms to information ecosystem theory, and the formation, dissemination, and guidance of news information in the network exhibit corresponding lifecycle characteristics [18]. Research on information lifecycle theory focuses on division of the information dissemination cycle and description of the pattern of public opinion evolution in each stage [19]. In existing studies, after induction, the life-cycle model of information is typically divided into four, five, or six stages [20]. Among them, the five-stage lifecycle model is the most common, containing the following stages: budding stage, spread stage, outbreak stage, recession stage, and long-tail stage (see Fig 2 for details). With the advent of the Internet and media convergence technology, a wide range of breaking news can be spread and forwarded rapidly [21]. Therefore, some scholars have sought to clarify the pattern of news information dissemination through the lifecycle model, to understand and guide the social impact of public opinion [22].

Recently, researchers have utilized information lifecycle theory to monitor public opinion regarding COVID-19 expressed on social media [23]. This approach is reported to be beneficial for understanding how to promptly direct public sentiment and, consequently, alleviate societal crises [24]. First, researchers analyze the variations in topic discussions on social media platforms across different countries, including language characteristics and repost frequency. This analysis can provide a micro-level perception of public attitudes at different stages of the pandemic, allowing for the deduction of the impact of COVID-19 on society. This information forms the basis for a comprehensive understanding of the developmental trends of information lifecycle theory and the exploration of the influencing mechanisms of internet discourse [25]. Furthermore, scholars have developed the pandemic information support lifecycle model on the basis of the crisis lifecycle model. This model offers a macro-level depiction of how specific information emerges, develops, and fades during a crisis, enhancing our ability to effectively track the changing trends in COVID-19-related social discourse [26].

Simultaneously, information lifecycle theory also offers theoretical guidance for governments’ approaches to managing public opinion [27]. Scholars have developed a visualization system for public opinion crises, which employs lifecycle theory to evaluate the current momentum of public opinion, enabling the identification of situations requiring government intervention to prevent the dissemination of false information and its adverse effects on the public in the online realm [28]. Currently, lifecycle theory is mainly applied in crisis management during emergencies, showcasing its wide-ranging prospects for studying the impact of COVID-19 news on social discourse [29]. Therefore, on the basis of the first three stages of lifecycle theory, in the present study, we focused on exploring the effects of news coverage concerning the initial wave of COVID-19 on the public and society. This study aimed to inform the provision of more precise strategies and approaches for managing public opinion crises [30].

### 2.2 Agenda-setting theory

With the continuous development of Internet technology and integrated media, news about emergency events can spread quickly through online media [31]. In emergencies like COVID-19, public sentiment can be particularly strongly affected by government news and media coverage. Hence, timely, objective, and impartial news reporting and public opinion guidance are essential for social stability [32]. According to agenda-setting theory, it can be difficult for the media to determine the public’s perception of an event, but they can influence public opinion about the facts and the order in which information is discussed by providing specific information or structuring the issue [33]. Agenda-setting can be divided into public agenda-setting, policy agenda-setting and media agenda-setting, distinguished by the different entities that determine the importance of issues [34]. The current study specifically focused on media agenda-setting, which refers to the influence of media on public attention and ideology. Agenda-setting theory suggests that the media not only informs individuals about how to think, but also guides their choices regarding which issues to focus on and contemplate [35]. In essence, the media can shape the public’s attention focus by selecting, emphasizing, and arranging relevant topics. Hence, the content and manner of news reporting shape the public’s understanding and the level of importance attributed to specific issues, thereby impacting public opinion on social topics [36]. Media agenda-setting theory examines the impact of media on social issues, and prompts reflection and discussion on the social responsibility of news media [37].

Recently, scholars have begun to apply agenda-setting theory to empirical analyses and case studies of media communication related to COVID-19 [38]. Existing studies have empirically analyzed the influence of discussions about trending COVID-19-related topics on social media platforms such as Twitter on users’ emotions. These studies have reported that social media has a stronger effect of guiding and shaping the cognition of younger individuals compared with older individuals, subsequently impacting their behavior [39]. To thoroughly investigate the impact of COVID-19-related news on various types of agenda-setting, scholars have utilized web crawling techniques to analyze COVID-19-related Facebook posts with big data analysis. By comparing public agenda-setting with media agenda-setting, research has revealed that news media posts tended to focus on describing and analyzing the developments and progress of COVID-19, whereas posts by members of the public tended to express personal feelings and emotions [40]. Public sentiment has been reported to change in response to media reports about COVID-19, further indicating that the media agenda and the public agenda influence each other [41].

Meanwhile, some scholars have also attempted to compare government-led and public-led agenda-setting through case studies to investigate whether the COVID-19 pandemic enhanced the government’s role in agenda-setting [42]. Case studies suggested that government-led and public-led agenda-setting were mixed during the COVID-19, with the Chinese government paying attention and responding to sentiments expressed by the public through social media [43]. On the basis of the analysis described above, the current study explored the impact of Baidu News headlines related to COVID-19 on social opinion and public sentiment using agenda-setting theory.

This study provides a brief review of recent literature on information lifecycle theory and agenda-setting theory, revealing that few studies have used these two theories to analyze the thematic characteristics, social network structure, and impact on public sentiment of COVID-19 epidemic-related news headlines. Therefore, to extend existing research findings, the current study provides a detailed discussion of the impact of Baidu News coverage of COVID-19 using lifecycle and agenda-setting theories, providing a scientific basis for epidemic-related public opinion and mental health management. Next, we conducted a theoretical analysis of the impact and management mechanisms of public opinion dissemination, and formulated specific research hypotheses.

## 3. Theoretical analysis and hypotheses

### 3.1 The impact mechanism

**Fig 3:**
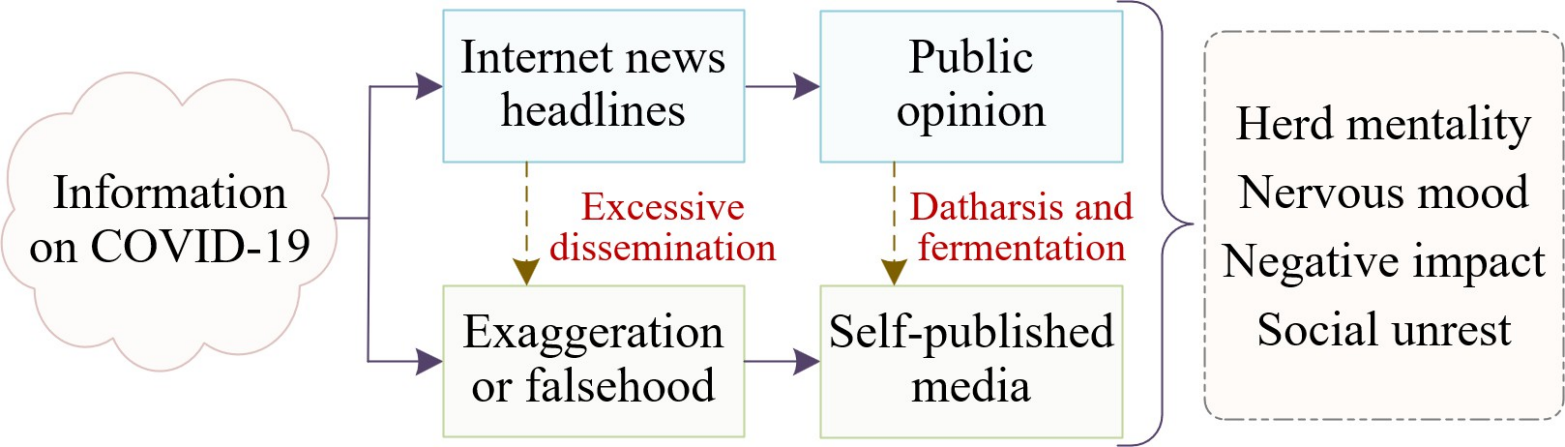
Impact mechanism of public opinion dissemination.

During the initial period of the COVID-19 outbreak, the news media emerged as a crucial force in shaping public discourse. Subsequently, on the basis of information lifecycle theory, we conducted an analysis of the impact mechanism by which news headlines trigger public opinion and shape public sentiment. The impact mechanism is illustrated in Fig 3.

First, the news media, as the dominant means of information dissemination, has had a profound impact on public awareness and understanding of COVID-19 [44]. The first wave of the COVID-19 epidemic had an uncertain origin and pathology. By reporting on the outbreak, the media initially communicated the seriousness of the situation, including the transmission routes and severity of COVID-19, prompting a shift in public attention from neglecting the virus to prioritizing the epidemic. However, the dissemination of such information can also lead to public panic and anxiety. Second, the news media has exhibited selective reporting when covering COVID-19. Some media outlets selectively highlighted the negative effects of the pandemic, deviating from the reality of the news events and hindering a comprehensive and objective depiction of the overall situation [45]. This can not only result in public misconceptions about the pandemic and trigger excessive levels of anxiety but can also have a subsequent impact on individuals’ attitudes and behaviors towards the outbreak. Finally, social media, as a highly interactive and rapid dissemination platform, also exerts a significant impact on public opinion, which should not be underestimated. During the initial stages of COVID-19, a substantial amount of information about the novel coronavirus circulated on social media, forming a self-sustaining information ecosystem. In such an information environment, individuals are susceptible to information overload, which can lead to feelings of panic [46].

Overall, the impact mechanism of news and public opinion during the early stages of COVID-19 was primarily manifested in the rapid guidance of public awareness about the epidemic through various information dissemination channels. Selective reporting by news media can lead to an imbalanced perception of the epidemic among the public, while rapid information dissemination on social media also creates pressure for the public. Therefore, it is important to recognize the impact mechanisms of emergencies like COVID-19 on public emotions and to strive to establish a scientific reporting mechanism to guide the public in understanding and responding to the epidemic appropriately. Thus, we formulated the following hypothesis:

**Hypothesis 1 (H1).** During the initial stage of public health emergencies, news headlines from various categories impact the level of public and societal attention toward relevant events.

### 3.2 The management mechanism

**Fig 4:**
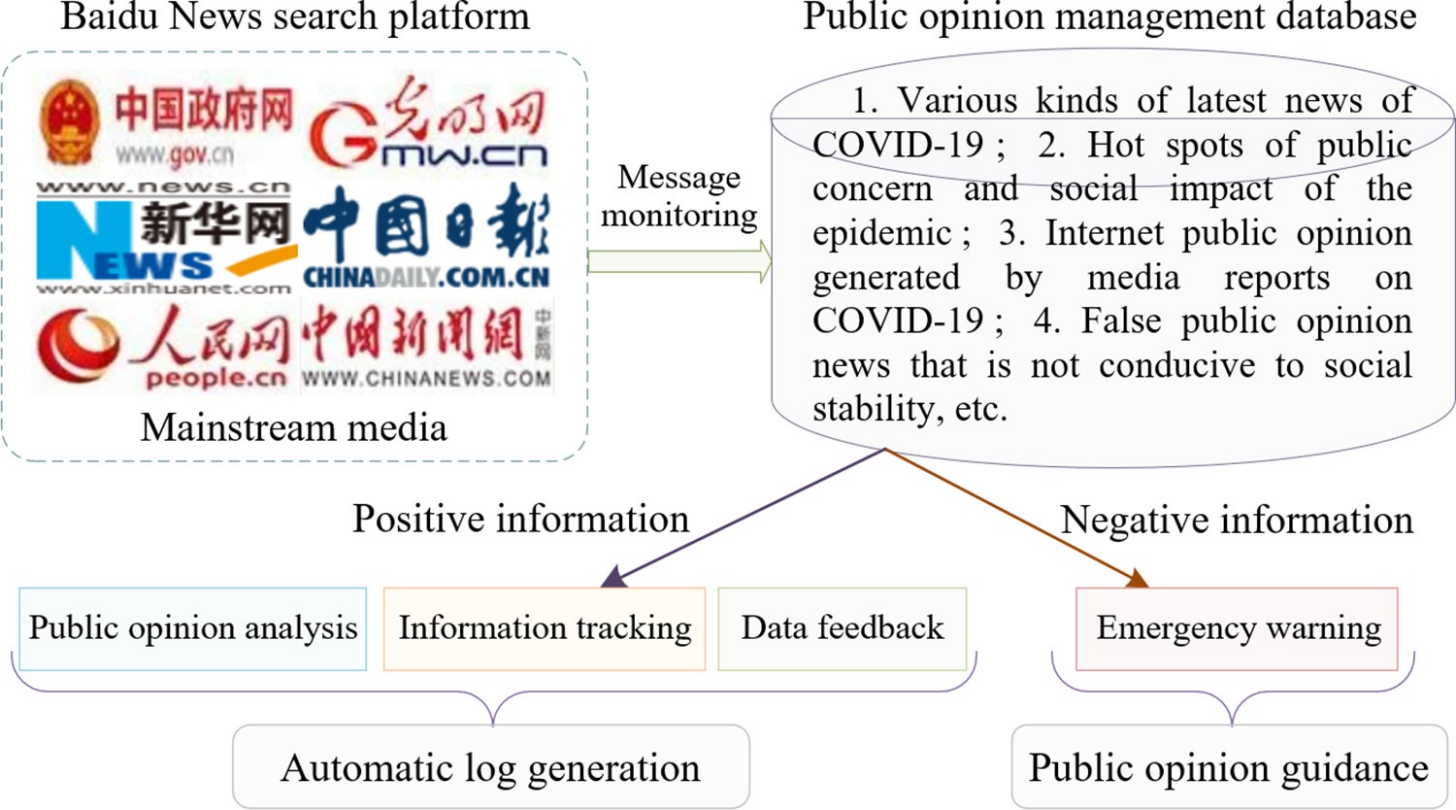
Management mechanism of public opinion dissemination.

During the COVID-19 epidemic, the spread of information has been closely connected to public opinion, and public sentiment has been greatly impacted by various forms of news media coverage. News media offers positive comprehension and guidance in combating the COVID-19 pandemic, while also generating negative impacts that lead to pessimistic emotions among the public. Given this situation, agenda-setting theory suggests that the news and public opinion management mechanism can effectively contain negative emotions among the public and prevent social crises. Next, we will explore the management mechanisms of news and public opinion in relation to three aspects: news dissemination, selective reporting, and social media. The management mechanism is depicted in Fig 4.

First, COVID-19 news headlines act as the initial basis of public sentiment, because news media offers the public the latest information. However, dissemination of exaggerated or false COVID-19 information can easily trigger social panic. To address this issue, scientific management mechanisms, including control and guidance, can ensure the accuracy and timeliness of news, reduce unfounded rumors and malicious hype, and guide the public in developing accurate perceptions of the COVID-19 situation [47]. Furthermore, news reports should include the latest developments on COVID-19, national prevention and control measures, and personal protection guidance. Moreover, news should promptly address the public’s doubts, allowing them to respond to COVID-19 rationally, on the basis of sufficient knowledge. Second, in terms of selective reporting, on the basis of agenda-setting theory, news organizations often prioritize news value and may emphasize the negative aspects of the epidemic. This selective reporting can magnify public feelings of panic. Management mechanisms can ensure balanced media reporting by highlighting not only the impact of the epidemic but also emphasizing preventive measures and their effectiveness [48]. Under the public opinion management mechanism, the news media ensures that the public is well-informed about the risks of COVID-19 and presents strategies for addressing it, motivating people to develop confidence in overcoming the virus. Finally, given the anonymity, interactivity, and broad reach of social media, it is crucial to scrutinize and filter the accuracy of information, because the spread of misinformation is strongly correlated with interactivity and anonymity. On the one hand, government departments and social media platforms need to collaborate to establish a strict information review system to verify the posted information and control malicious dissemination [49]. On the other hand, social media platforms can create specialized channels for the epidemic, where official accounts should share authoritative and accurate COVID-19 information in a timely way, facilitating public access [50].

In general, the management mechanisms of news and public opinion aim to achieve balanced dissemination of information and ensure fairness and accuracy in the content being communicated. We believe that utilizing the media in a reasonable manner can effectively guide public emotions during the COVID-19 epidemic. Therefore, public opinion management should not only harness the social responsibility of the news media but also enhance its capacity to guide public opinion, serving COVID-19 prevention and control and the psychological well-being of the public. Thus, we formulated the following hypothesis:

**Hypothesis 2 (H2).** An improved public opinion management mechanism can effectively alleviate public sentiment and mitigate social crises.

## 4. Data and methods

### 4.1 Data sources

To accurately explore Internet-based public opinion dissemination and public impact in the initial stage of the epidemic, the current study examined Baidu News headlines during the first wave of COVID-19 as the analysis sample. There were three reasons for this approach. First, Baidu News is not only the largest Chinese search engine, but also the largest mainstream news reporting channel in China. Baidu News responds to billions of Chinese news search requests every day, and most important Chinese news articles related to COVID-19 can be searched through Baidu News. Thus, Baidu News is an important tool for researching epidemic-related news in China. Second, other news portals, such as Sina News and China Daily, mainly function to browse news within their websites, and the ability to search for news using these platforms is weak. In contrast, Baidu News has a powerful search function, which enabled us to use crawler technology to obtain comprehensive information about the epidemic, and to conduct data analysis. Finally, the credibility and influence of mainstream media creates its authority, and is also directly related to the impact of the media on society. Social media or self-published media coverage of the epidemic may be superficial, short-lived and low in readership, whereas mainstream media coverage of the epidemic plays an important role in guiding the formation and development of social opinion.

The specific process of collecting sample data is described below. We searched Baidu News using the keywords “Novel Coronavirus” and “COVID-19” from 8 January to 21 February 2020 (45 days in total) and selected “time order” and “all information” in the search. In total, 1,076 Baidu News headline samples were identified. Although the number of samples was limited, the news in the sample was released by official or mainstream media, and the information density was high. Thus, this sample is likely to reflect the trends in the development of the epidemic and the direction of social opinion. We then used Python V3.6 crawling to capture key information, such as the title, summary, released media, and release time for each COVID-19 news item. To facilitate result reproducibility, we have uploaded the data and code for this study as “Supporting information” (S1 File), and it can also be accessed through https://protocols.io/view/plosone-c5ify4bn.

### 4.2 Sample description

In the early stages of the epidemic, because the cause of unknown pneumonia, as well as the type and source of the virus, were not well understood, the media referred to the epidemic inconsistently, using the terms “novel coronavirus” and “unknown pneumonia” to refer to it in most headlines. As searched in Baidu News, on 8 January 2020, the mainstream media reported that a team of medical experts had found an unknown type of viral pneumonia infection in Wuhan. Subsequently, there was widespread human-to-human transmission of “Wuhan pneumonia,” which was identified by authoritative medical experts as being caused by a “novel coronavirus.” On 11 February 2020, when cases of the novel coronavirus were found in several countries around the world, the World Health Organization officially named the pneumonia infected by the novel coronavirus “COVID-19,” at which point COVID-19 became the official term.

Therefore, the terms “Wuhan pneumonia,” “unknown pneumonia,” and “novel coronavirus,” which appeared in many news articles, all refer to “COVID-19” in essence. To ensure that the examined subject terms conformed to standard international terminology, and to enable more accurate clustering of the sample data in social network analysis, we grouped related subject terms, such as “unknown pneumonia” and “novel coronavirus,” with the term “COVID-19.”

Because of the rapid spread of the COVID-19 epidemic, it took only 45 days for Internet headlines related to the epidemic to move from a budding stage to a full-blown outbreak, attracting a high level of attention from government, media, and the public. Prior to 8 January 2020, there were relatively few cases of unknown pneumonia, so there was very little related news and limited public attention. When human-to-human transmission was reported to have occurred, the public began to pay attention to the development of the outbreak. At this point, public opinion began to form, and the budding stage of the information lifecycle began. By 21 February 2020, with the gradual expansion of the impact of COVID-19 and the adoption of strict control measures in many places to contain the development of COVID-19, the general public were highly concerned about the development of the epidemic. At this point, the number of related news items increased rapidly, and a long-lasting outbreak stage began. Therefore, in the current study, we set the time span of the Baidu News headlines sample to the period from 8 January to 21 February 2020.

### 4.3 Word segmentation

After sample capture, the news data were structurally processed via manual cleaning, and information that was not relevant to COVID-19 was removed, such as symbols, pictures, videos, or websites. We used the word segmentation function of ROST CM V6.0 to sort the information abstracts. We added new words, including “COVID-19,” “epidemic situation,” “unknown pneumonia,” and “suspected” in the word segmentation table, and completed the steps of adding high-frequency new words, deleting invalid words, and merging similar words. Table 1 shows the segmentation results for COVID-19-related Baidu News headlines.

**Table 1:** Partial results of COVID-19 news headlines after word segmentation.

S.N.	Baidu News headlines	Results after word segmentation	Source	Release time
1	Chunlan Sun is in Wuhan to investigate the prevention and control of the novel coronavirus pneumonia epidemic.	The State Council; Vice-premier; Hubei Province; Wuhan City; Inspection guidance; Novel coronavirus; Pneumonia; Epidemic prevention and control; Take strong measures; Effective ways	gov.cn	22:50 Feb 10, 2020
2	The Health Commission will launch an inter-provincial matching support program for all provinces in Hubei except Wuhan.	Resolutely implement; the State Council Decision making and deployment; Full support; Hubei Province; develop; novel coronavirus Prevention and treatment of pneumonia; Be determined to win; Epidemic prevention and control	news.cn	9:17 Feb 11, 2020
3	The World Health Organization reports that the novel coronavirus is related to the Bat Chrysanthemum coronavirus.	World health organization; WHO; release; novel coronavirus; Report display; Bat transmission; 2019-nCoV; Connected; Clinical treatment; Patient in severe condition	cctv.com	13:34 Feb 12, 2020
4	What have we learned from the novel coronavirus epidemic?	Sudden; COVID-19 Epidemic situation; Influence; Hundreds of millions of people; World attention; WHO; Novel coronavirus infected; Pneumonia epidemic situation; International emergency; Public health events	81.cn	16:52 Feb 14, 2020
5	SINOPHARM: A total of 1 million copies of the novel coronavirus test kit have been produced.	China; Pharmaceutical Group; Understand; COVID-19; Epidemic situation; Enterprise; Cumulative production; Novel coronavirus; Nucleic acid test; Test kit; 1 million copies	people.cn	18:39 Feb 15, 2020
6	“Anti-epidemic” animated propaganda film “The Novel Coronavirus Prevention Notice”.	Novel coronavirus; An infection; Pneumonia; The epidemic; To upset; Life; The rhythm; Novel coronavirus; Dialogue; Screenshots; Photos; Network; The media; Socialize; Platform; One after another; It’s hard to tell the truth from the fake.	gmw.cn	14:07 Feb 16, 2020

### 4.4 Word frequency statistics

**Fig 5:**
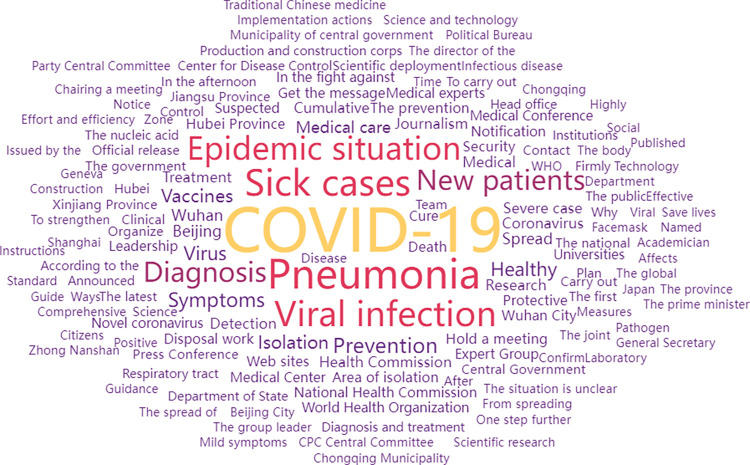
Word cloud of high-frequency words in COVID-19-related news.

We used ROST CM V6.0 to construct a “COVID-19” corpus of 1,076 standard sample data points after word segmentation and conducted keyword frequency analysis (see Table 2). We then used WordCloud to generate a word cloud (Fig 5) to highlight information topics. As shown in Fig 5, all keywords radiated outward around “COVID-19,” “Pneumonia,” “Sick cases,” “Viral infection,” and “Epidemic situation,” indicating that early COVID-19 news covered a wide range of key topics and had a strong hierarchy.

**Table 2:** The 15 leading high-frequency keywords in COVID-19 news.

Keywords	Word frequency	Keywords	Word frequency	Keywords	Word frequency
COVID-19	1184	New patients	438	Symptoms	97
Pneumonia	926	Diagnosis	332	Vaccines	86
Sick cases	761	Prevention	242	Isolation	75
Viral infection	538	Healthy	167	Medical care	71
Epidemic situation	481	Virus	105	Wuhan	64

**Fig 6:**
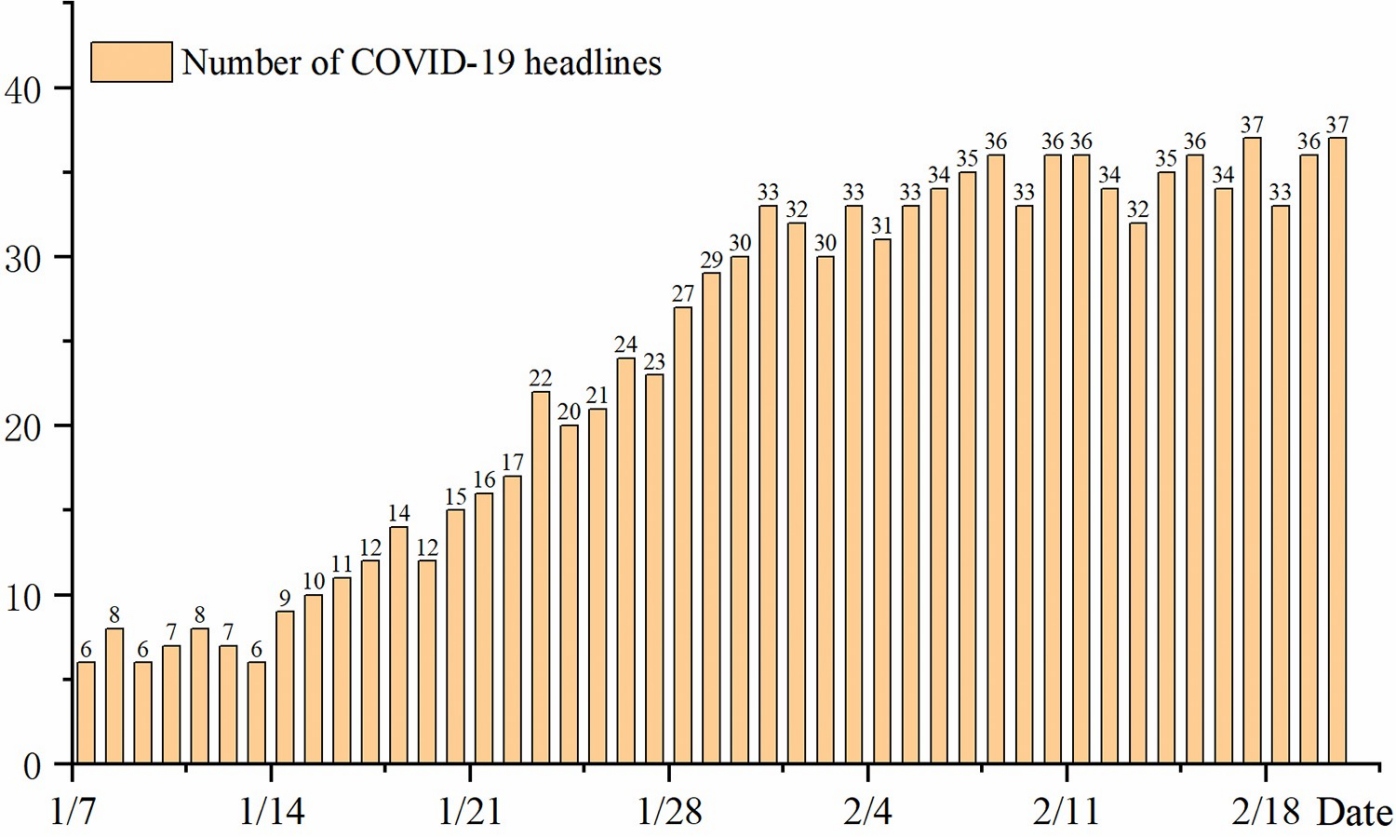
Number of daily news headlines about COVID-19 in Baidu News.

**Fig 7:**
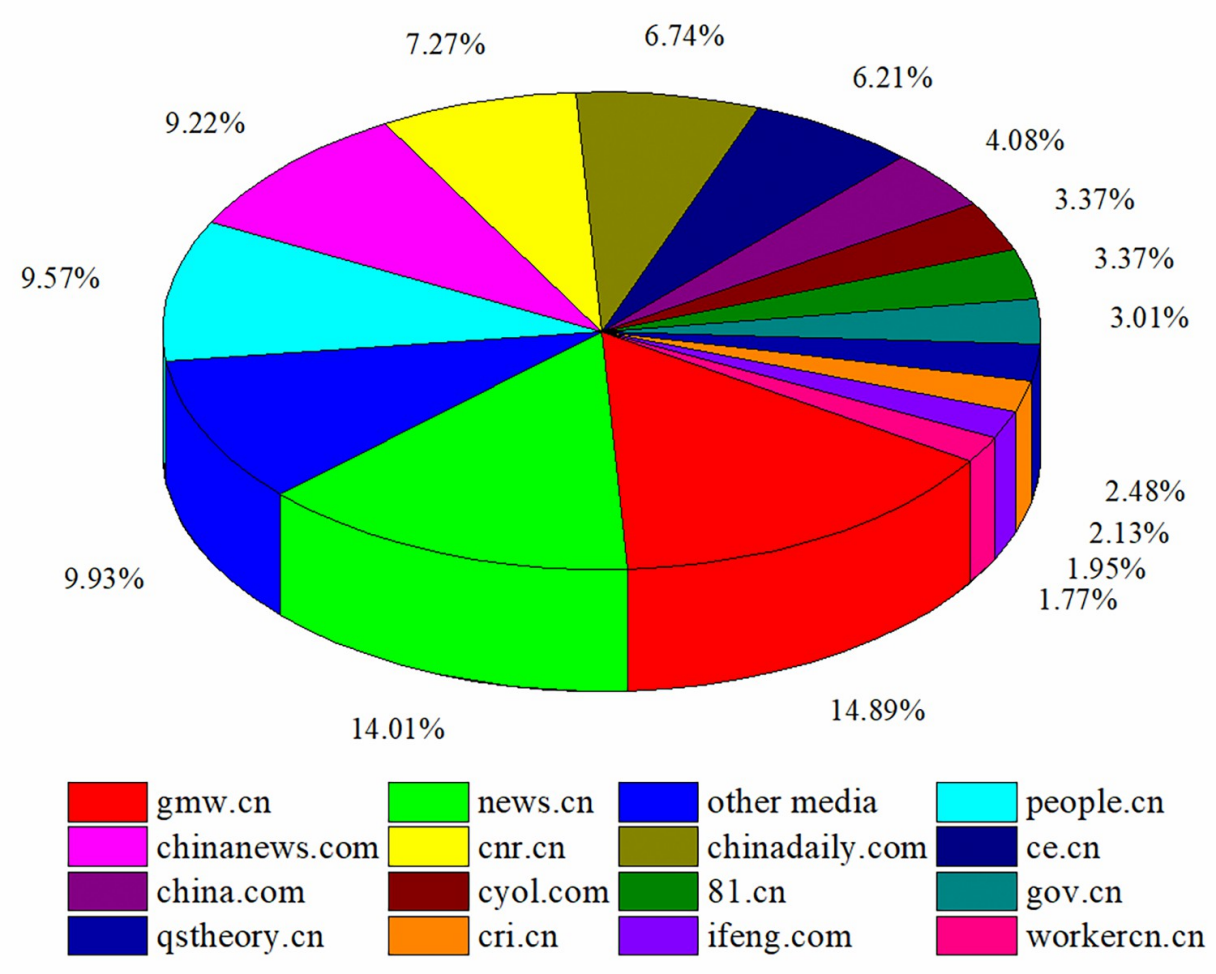
Proportion of media outlets reporting COVID-19 news.

Figs 6 and 7 were created using OriginPro V2022 and statistics on the amount of information in the corpus and published media. Fig 6 shows that there was little information about COVID-19 released by Baidu News from 8 to 20 January 2020. The only reported news indicated that an unknown virus that caused pneumonia was found at the Wuhan seafood market, but that no human-to-human transmission had been found. Therefore, this news coverage did not attract public attention, and represented the budding stage of the lifecycle. On 21 January 2020, human-to-human transmission of an unknown type of pneumonia was reported. News reports also stated that patients with COVID-19 were identified in many parts of the country and large numbers of doctors and nurses were infected. At this time, the news reporting on COVID-19 entered the outbreak period and continued to grow, becoming the focus of social attention. Examining the information lifecycle model (Fig 2), we can infer that the epidemic information was in the stage of rapid outbreak at this point, and the news that “COVID-19 can spread among different people” was the outbreak point of public opinion.

Fig 7 shows that online news media outlets with more than 50 news releases about COVID-19 in the sample were as follows: gmw.cn (84), news.cn (79), people.cn (56), chinanews.com (54), and cnr.cn (52). These five websites are the most influential official media outlets in China, playing the role of “opinion leaders.” These opinion leaders possessed the most COVID-19 information resources, and were at the core of the dissemination of information about the epidemic. According to agenda-setting theory, the powerful influence of mainstream media can curb the spread of rumors and promote the development of positive public opinion.

### 4.5 Main methods

Informetrics is a useful method for studying the overall structure and development trends in public emergencies [51]. Informetrics includes scene visualization according to time series and social network analysis on the basis of co-word relationships [52]. In this study, we used Baidu News headlines regarding COVID-19 as a sample. We then crawled and segregated Baidu News information during the first COVID-19 epidemic wave, gathered information topics using cluster analysis, explored the evolutionary process of epidemic information on the basis of time-series analysis, and measured node centrality and structural holes using social network analysis.

1. Cluster analysis is a research method for classifying inquiry objects according to sample characteristics. Cluster analysis aggregates closely related sample data with similar characteristics so that all variables can be clustered into a classification system from micro to macro. In this method, a pedigree network or thermal diagram is used to represent the relationships between samples. In the current study, VOSviewer V6.19 was used to cluster the sample data and generate a visual network and thermal diagram [53]. Nodes with the same color represent the same category. A large node size indicates that the keywords appear frequently and are in a core position. A thick line indicates that two words have more co-occurrences and are closely related.
2. Timeline evolutionary analysis is derived from clustering analysis. Each clustering keyword is marked with time to outline the time series of nodes within the cluster and the time span between topic clusters [54]. In this study, we used VOSviewer V6.19 for time evolution analysis to clearly show the relationship between nodes in a certain time span on the basis of tag clustering.
3. Co-word analysis refers to co-presentation of the title, author, abstract, and unstructured high-frequency keywords and phrases of text data to reflect the relevant strength of text content, then determining information hotspots, paradigms, and composition represented by these words [55]. We used ROST CM V6.0 to count the frequency of subject words in the same kind of text. Additionally, we used Bibexcel V2016 to form a co-word matrix and a similarity matrix of associated words and judge the relevance of subject content using the distance between network nodes.
4. We used social network analysis to calculate the special associations among nodes in a social network system through data mining, and displayed the characteristics of the network model using Ucinet V6.0 [56]. The social network was calculated using the following parameters. Group clustering refers to the use of K-means to classify and count high-frequency words according to attributes and characteristics, and distinguish the differences between word clusters. Centrality, which includes degree centrality, betweenness centrality, and closeness centrality, is a measure of the centrality of social networks. Structural holes refer to the non-redundant connections between two key word nodes and can provide advantages in information dissemination to nodes.

## 5. Results

### 5.1 Visualization analysis

#### (1) Cluster visualization

**Fig 8:**
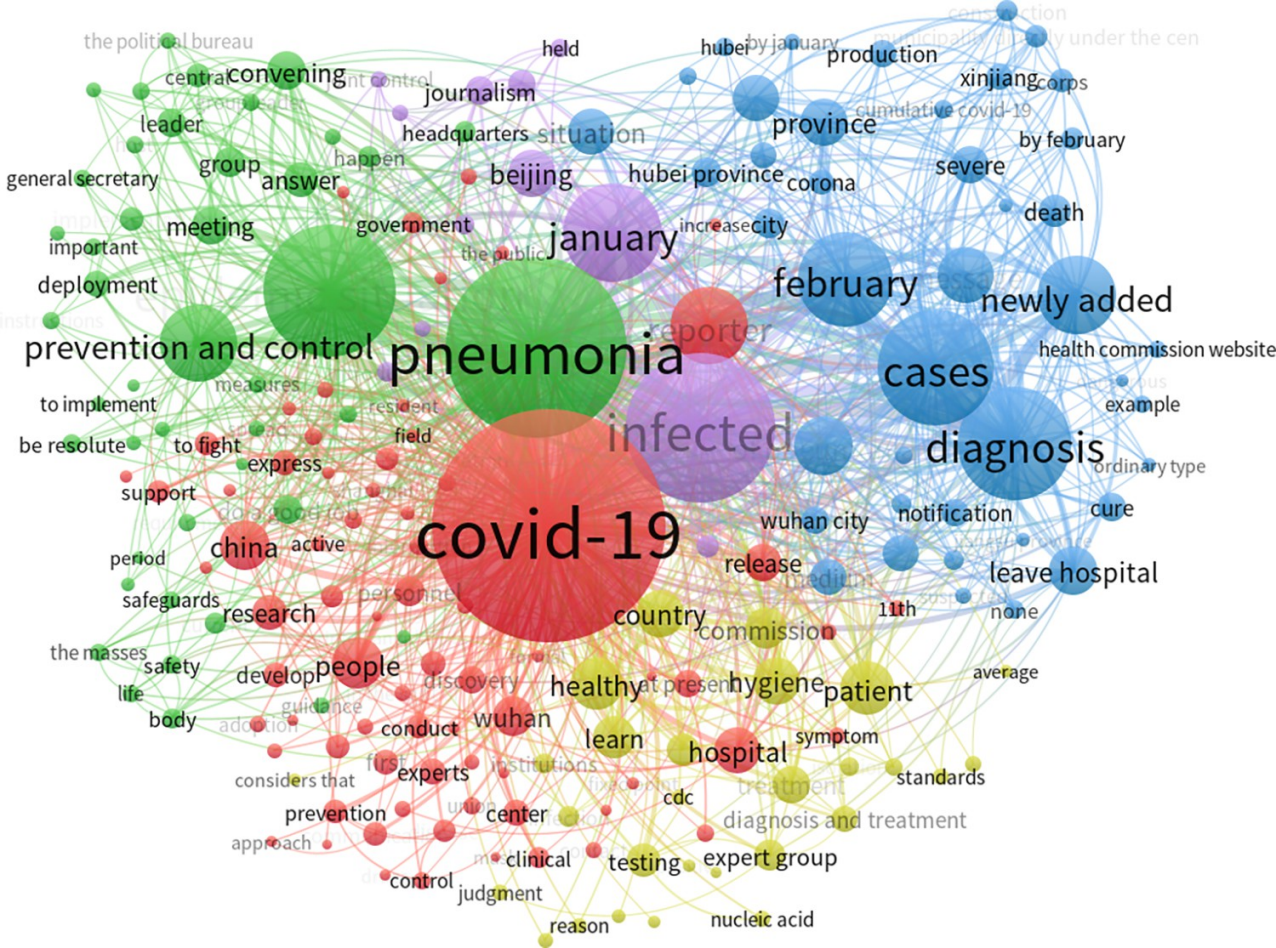
Clustering network of high-frequency words in COVID-19-related news.

**Fig 9:**
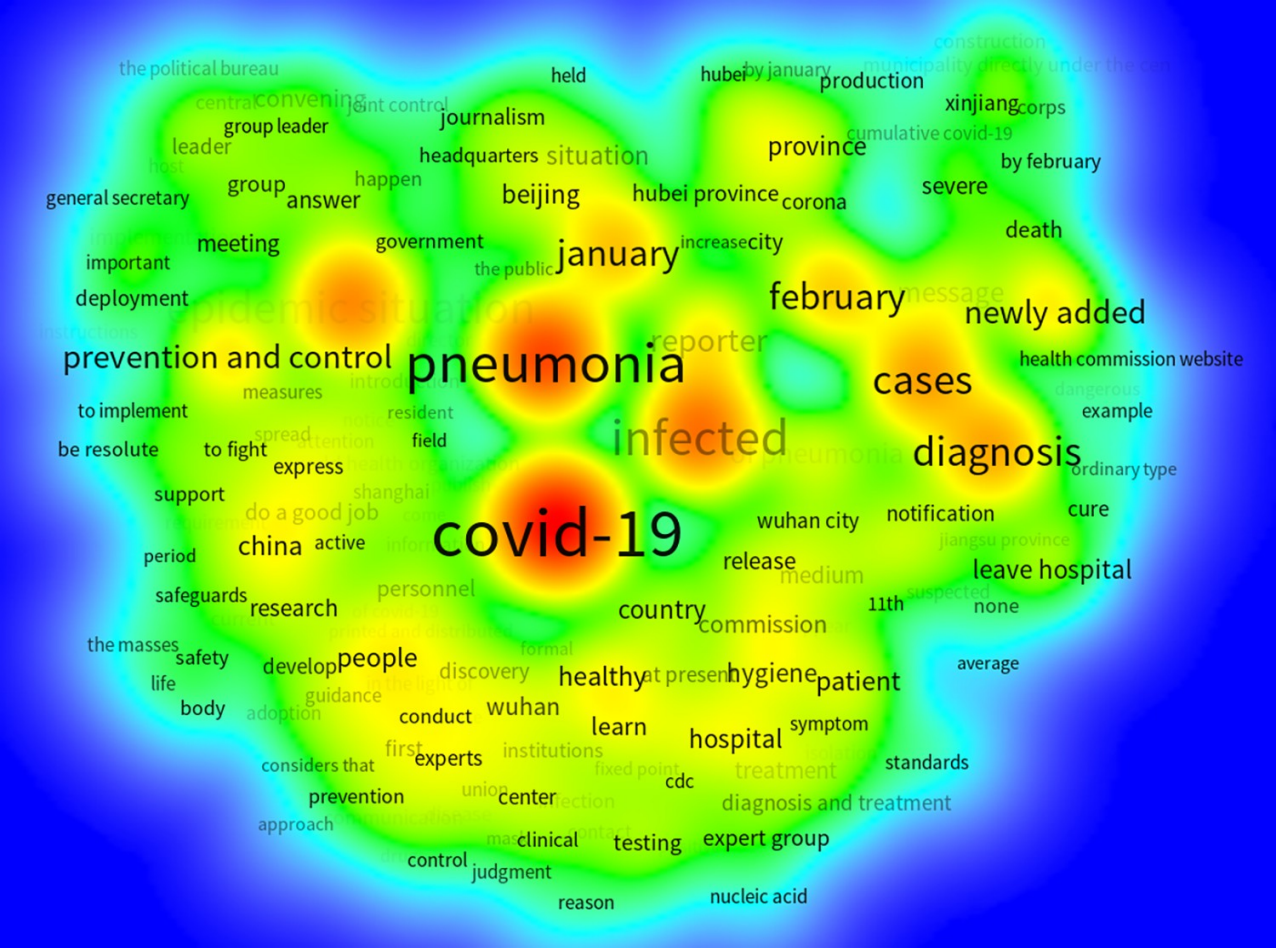
Thermal diagram of high-frequency words in COVID-19-related news.

To accurately analyze the timeliness of COVID-19 news-related public opinion and the impact of news headlines on public attention, we included time-series factors in a cluster-view analysis of high-frequency words [57]. In this study, VOSviewer V6.19 was used to extract COVID-19 news keywords and word frequency from the sample. We extracted 156 keywords with more than 15 frequencies and practical significance. The co-occurrence frequency was used as the weight value to weigh the nodes and connections, and the clustering networks (Fig 8) and thermal diagram (Fig 9) were fitted using the Modularity option.

In the clustering network graph, nodes with the same color represent the same category. A large node size indicates that the keywords appear frequently and are at the core of the network. A thick line indicates that the two words have more frequent occurrences and are closely related. In Fig 8, keywords are classified into five categories. The two keywords “COVID-19” and “Pneumonia” are at the core of the node whereas the nodes “Viral infection” and “Sick cases” are slightly lower. “Epidemic situation,” “Beijing,” and “Prevention and control” are close to the core node, and other high-frequency words extend outward with the core keyword as the center. Among them, “COVID-19,” “Viral infection,” and “Sick cases” were the most closely related; “Pneumonia,” “Epidemic situation,” and “Prevention and control” were closely related; and the connection density of peripheral keywords and core keyword nodes was not high, and was roughly similar. The theme density of the thermal diagram (Fig 9) depends on the weight value of a node and its surrounding nodes. Brighter color indicates greater heat, weight, and frequency of a keyword. This indicates that news headlines from different categories can impact the level of public attention towards relevant events. Through media coverage, the public shifted from being uninterested to highly attentive, supporting hypothesis H1.

#### (2) Time-series visualization

**Fig 10:**
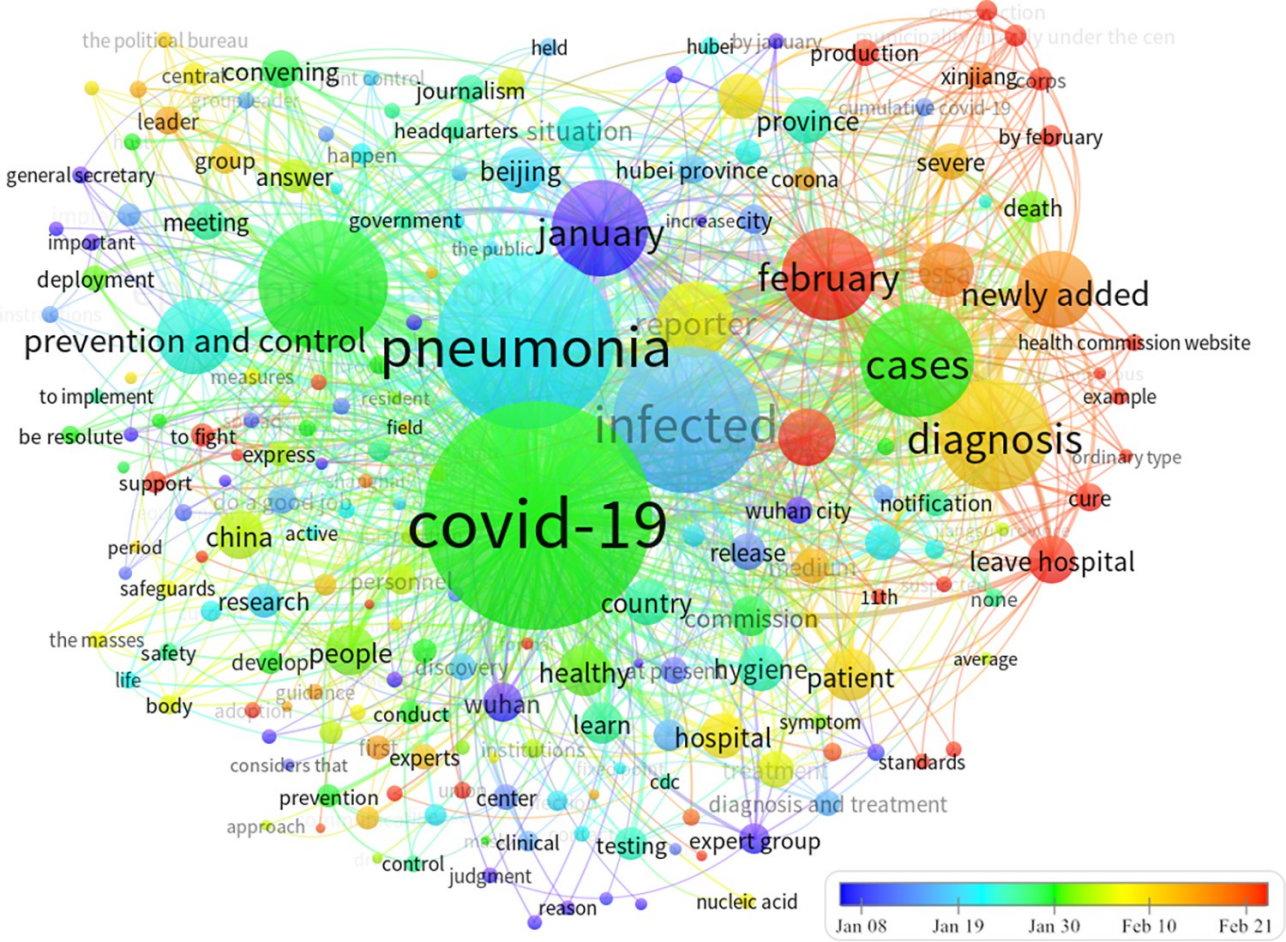
Time co-occurrence network of high-frequency words in COVID-19-related news.

A time co-occurrence network graph (Fig 10), spanning from 8 January to 21 February 2020, includes a time series that is based on keyword co-occurrence and marks the occurrence time of keywords using different node colors [58]. In Fig 10, “Expert group,” “Wuhan,” “Infection,” and other keyword nodes appear in blue, indicating that these keywords first appeared on 8 January 2020. The “COVID-19,” “Pneumonia,” and “Epidemic situation” nodes are green and appeared on 30 January 2020. The “Cure,” “Recovery,” and “Leave hospital” nodes are red and appeared on 21 February 2020. These findings indicate that the extracted high-frequency news keywords were consistent with the timeline of COVID-19 epidemic development.

On the basis of these results, we can infer the propagation process of the first wave of COVID-19 news-related public opinion. The approximate timeline of this process was as follows: (1) The expert group identified cases of an unknown type of viral pneumonia and isolated infected patients in the hospital. (2) The State Council held a press conference to report on this unknown pneumonia and directed the deployment of prevention and control measures. (3) The National Health Commission reported on infection, prevention, and control of this novel pneumonia in Hubei Province. (4) The Chinese government’s action against COVID-19 received the attention and support of various countries, and the World Health Organization. (5) The rate of increase in the number of confirmed cases among suspected cases began to decline, and patients were cured and discharged from hospital. Hypothesis 1 was further supported, confirming that news reporting impacted the level of public attention toward the public health emergency.

### 5.2 Social network analysis

#### (1) Constructing the co-word matrix

Co-word matrix methods can be used to analyze high-frequency keyword logic and identify similarities. In this approach, an equivalence coefficient is used to cluster keywords and convert them into a two-dimensional matrix. The process of conversion weakens the adverse factors that significantly reduce measurement accuracy owing to differences in word frequency between keywords [59]. We used the Co-occurrence function in Bibexcel V2016 to transfer high-frequency keywords to a 158 × 158 co-word matrix of epidemic information for social network analysis. Owing to space limitations, Table 3 only shows the co-word matrix for the top 10 high-frequency keywords.

**Table 3:** Co-word matrix of 10 leading high-frequency keywords.

Keywords	COVID-19	Pneumonia	Sick cases	Viral infection	Epidemic situation	New patients	Diagnosis	Prevention	Healthy	Virus
**COVID-19**	1184	615	481	315	248	179	187	153	102	144
**Pneumonia**	615	926	394	267	179	146	169	106	76	97
**Sick cases**	481	394	761	346	80	135	171	45	53	57
**Viral infection**	315	267	346	538	160	97	220	101	68	106
**Epidemic situation**	248	179	80	160	481	268	155	146	43	83
**New patients**	179	146	135	97	268	438	216	119	46	44
**Diagnosis**	187	169	171	220	155	216	332	164	41	45
**Prevention**	153	106	45	101	146	119	164	242	128	49
**Healthy**	102	76	53	68	43	46	41	128	167	86
**Virus**	144	97	57	106	83	44	45	49	86	105

#### (2) Social network co-occurrence

**Fig 11:**
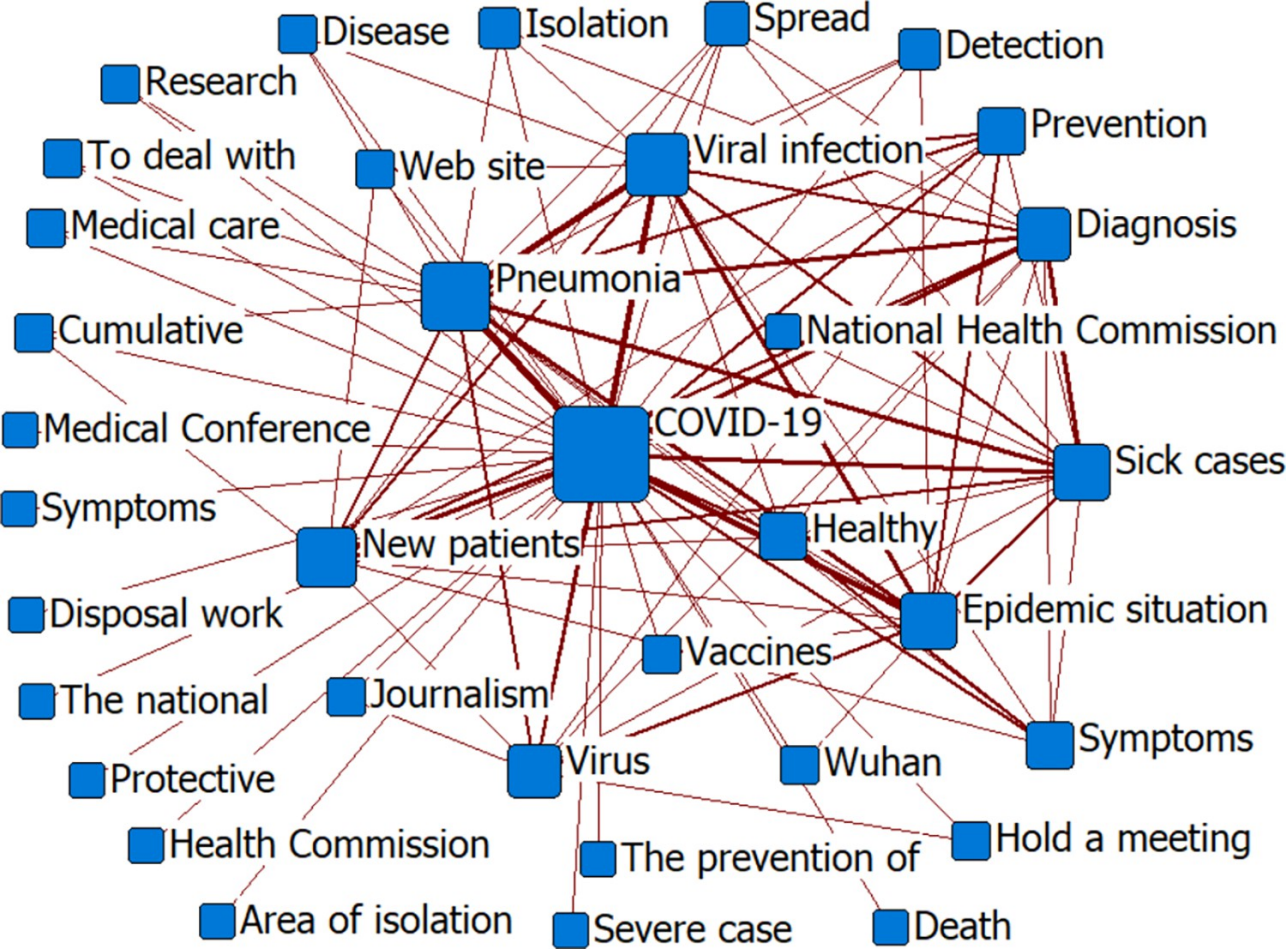
Social network of COVID-19-related news.

**Fig 12:**
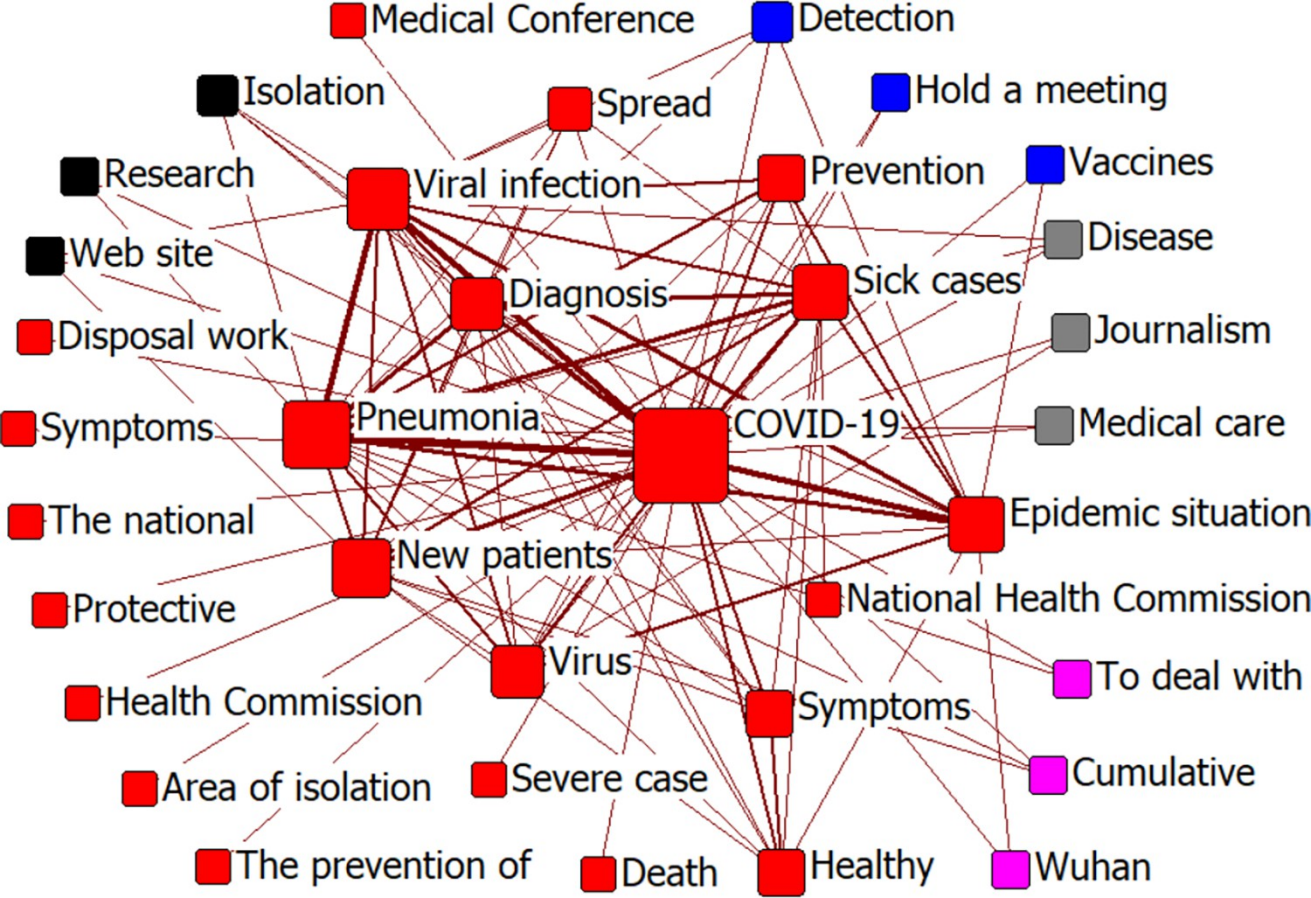
Group clustering of COVID-19-related news.

We imported the co-word matrix into Ucinet V6.0 and used the Netdraw option to draw a social network map of COVID-19 news information [60]. First, we selected “Degree” in the node type to analyze the co-occurrence of high-frequency words and generate a social network map (Fig 11). We then used the Factions function to conduct small group analysis and generate a group clustering diagram (Fig 12). Finally, we calculated the total density of the social network.

A large node size in the figure indicates that the keyword frequency is high, and a thick line indicates that the co-occurrence frequency of two words is strong. In Fig 11, the node “COVID-19” shows the highest frequency for this keyword. The connection between nodes, such as “COVID-19,” “Pneumonia,” “Viral infection,” “Vaccines,” “Epidemic situation,” and “New patients” is represented by a thick line, indicating that the above keywords were closely related. In Fig 12, high-frequency keywords are clustered into five groups. The representative groups are “COVID-19, Pneumonia, Diagnosis, Symptoms;” “Hospital, Medical care, Treatment, Healthy;” and “Beijing, Wuhan, Hubei Province.” We calculated the average density of the social network, which was 0.64 with a standard deviation of 9.15. The network density among these groups was close to 0.5, indicating that the social network of epidemic information was complete and node information mobility was strong. A large standard deviation indicates that initial news regarding the COVID-19 epidemic fluctuated over a wide range and covered a wide range of fields.

#### (3) Social network centrality analysis

The co-word matrix was imported into Ucinet V6.0, and centrality was measured using the Centrality option of the Network function [61]. The specific results of the three types of centrality (Table 4), and descriptive statistics of network centrality (Table 5) were obtained. As shown in Table 4, the three nodes “COVID-19,” “Pneumonia,” and “Viral infection” had the highest degree of centrality and betweenness centrality and the lowest closeness centrality, indicating that these three high-frequency keywords occupied the dominant position in the social network, and “COVID-19” was in the absolute core position of the social network. Combining the centrality of high-frequency words with the descriptive statistics of centrality in Table 5, we found that the degree centrality of 19 keywords, including “Pneumonia,” “Viral infection,” “Sick cases,” “Epidemic situation,” and “Diagnosis,” was greater than the average value of 100.57, indicating that these keywords were in a key position of the network and that they appeared frequently in COVID-19 epidemic-related news. The closeness centrality of 12 keywords, including “Pneumonia,” “Viral infection,” “New patients,” “Sick cases,” and “Epidemic situation,” was less than the average value of 19,497.72, indicating that these keywords were used as intermediary points of shorter paths to associate with other nodes. The betweenness centrality of the six keywords “Pneumonia,” “Sick cases,” “Viral infection,” “Epidemic situation,” “New patients,” and “Diagnosis” was greater than the average value of 3.29, indicating that these keywords were used as bridges to connect with other nodes, which can be summarized as hot spots of COVID-19-related news dissemination. This finding suggests that government departments should effectively regulate these key terms in news headlines to steer public opinion and mitigate the negative impact on society. Thus, the results suggest that an improved management mechanism could potentially alleviate public emotional responses and ameliorate social crises, supporting Hypothesis 2.

**Table 4:** Network centrality of some high-frequency keywords.

Degree centrality			Closeness centrality			Betweenness centrality		
Keyword	Degree	Nrm Degree	Keyword	Farness	N Closeness	Keyword	Betweenness	N Betweenness
COVID-19	3126.00	5.455	COVID-19	19469.00	0.806	COVID-19	396.079	3.234
Pneumonia	2044.00	3.567	Pneumonia	19484.00	0.806	Pneumonia	39.745	0.325
Viral infection	1669.00	2.912	Viral infection	19487.00	0.806	Viral infection	34.829	0.284
Sick cases	1243.00	2.169	New patients	19489.00	0.806	Sick cases	15.745	0.129
Epidemic situation	1201.00	2.096	Sick cases	19490.00	0.806	Epidemic situation	12.702	0.104
Diagnosis	1037.00	1.810	Epidemic situation	19490.00	0.806	New patients	8.995	0.073
New patients	1024.00	1.787	Diagnosis	19491.00	0.805	Diagnosis	8.643	0.071
Prevention	717.00	1.251	Healthy	19494.00	0.805	Healthy	3.119	0.025

**Table 5:** Descriptive statistics of network centrality.

Index	Degree centrality		Closeness centrality		Betweenness centrality	
	Degree	Nrm Degree	Farness	N Closeness	Betweenness	N Betweenness
**Mean**	100.570	0.175	19497.715	0.805	396.079	3.234
**Std Dev**	372.648	0.650	8.193	0.000	39.745	0.325
**Sum**	15890.00	27.729	682420.000	28.183	34.829	0.284
**Variance**	138866.9	0.423	67.118	28.183	1003.381	0.067

#### (4) Structural holes analysis

We used the Structural holes function in Ucinet V6.0 to calculate the structural holes parameters [62]. The results are shown in Table 6. According to structural holes theory, as the effective size parameter increases, the node is more likely to be at the core of the network. Among the nodes, the parameter value of “COVID-19” was the largest, followed by the parameter values of “Pneumonia,” “Viral infection,” “New patients,” and “Epidemic situation,” indicating that these keywords have large non-redundant factors in the network and are at the core of the network. On the basis of the efficiency value, the parameter values of “COVID-19,” “Pneumonia,” “Virus,” “Epidemic situation,” and “New patients” were large. From the perspective of the degree of constraint, “COVID-19,” “Pneumonia,” “Viral infection,” “New patients,” and “Sick cases,” are the first five nodes with the lowest degree of constraint, indicating that they have a strong ability to use structural holes. From the perspective of the hierarchy, “COVID-19,” “Pneumonia” “Viral infection,” “Epidemic situation,” and “Sick cases,” have a higher grade index, indicating that these keywords are at the core of the social network, have stronger control over the network than edge nodes, and occupy more structural holes. These findings suggest that it is important for public opinion management departments to effectively regulate the keywords that occupy central positions in structural holes, particularly focusing on the dissemination and comments related to these keywords in news headlines, to ensure social order stability. Thus, Hypothesis 2 is further supported. Additionally, the findings above indicate that the structural holes analysis results in the social network were highly similar to the clustering visualization results obtained using VOSviewer V6.19, further verifying the validity of the study methods.

**Table 6:** Statistics of structural holes parameters for the 10 leading keywords.

Keywords	Degree	Effective size	Efficiency	Constraint	Hierarchy
COVID-19	34.000	25.328	0.745	0.253	0.462
Pneumonia	19.000	10.766	0.567	0.325	0.327
Sick cases	13.000	6.781	0.522	0.372	0.217
Viral infection	16.000	8.447	0.528	0.344	0.276
Epidemic situation	13.000	7.051	0.542	0.386	0.243
New patients	14.000	7.506	0.536	0.359	0.214
Diagnosis	12.000	5.866	0.489	0.393	0.186
Prevention	8.000	3.606	0.451	0.507	0.105
Healthy	9.000	4.421	0.491	0.439	0.070
Virus	11.000	6.028	0.548	0.405	0.163

## 6. Discussion

On the basis of the results, we discussed three research implications.

1. Media websites should standardize the release of information via an audit mechanism. During the first wave of COVID-19, a substantial amount of unconfirmed information was spread via networks without being verified, which influenced public opinion and led to the Internet becoming a hotbed for the spread of false information. When publishing and updating news, the official media should follow scientific and standardized methods to curb the spread of false information. Portal websites should establish dynamic epidemic situation tracking platforms, disseminate accurate and relevant information, and prevent the public from being misled by false information.
2. National Cyberspace Administration should establish a long-term mechanism for public opinion early warning and management. In the initial stages of COVID-19, uncertainty about the development of the epidemic led to negative emotional responses among the general public. With a change or even reversal in public opinion hot spots and exaggeration in some news reports, the likelihood of rumors increased. When relevant agencies detected the large-scale spread of false information, it should issue emergency announcements to dispel rumors using its authority as an official media source to ensure the psychological well-being of the public.
3. Government departments should build two-way information communication mechanisms. Internet-based media facilitates public access to information, but one-way transmission of information may cause public anxiety. Therefore, establishment of two-way “official-public” communication mechanisms could create an important link that may aid in successful COVID-19 epidemic management. Government departments should aim to obtain information regarding public demands to ensure timely understanding of the public’s needs and accurate implementation of policies.

## 7. Conclusions

In this study, we used Baidu News headlines during the first wave of the COVID-19 epidemic as research data to analyze the lifecycle and evolution of information during a public health emergency. We crawled 1,076 news items about COVID-19 identified in a search of Baidu News from 8 January to 21 February 2020. We conducted word separation and quantitative statistics on news headline features, such as keywords and media sources for the sample. A co-word matrix was also constructed, and time series analysis as well as social network analysis were conducted on the basis of topic word clustering. In this section, we summarize the findings and limitations of the current study. Table 7 shows the research hypotheses and a summary of the results.

**Table 7:** Research hypotheses and its results.

S.N.	Hypotheses	Theories	Results
H1	During the initial stage of public health emergencies, news headlines from various categories impact the level of public and societal attention toward relevant events.	Information lifecycle theory	Valid
H2	An improved public opinion management mechanism can effectively alleviate public sentiment and mitigate social crises.	Agenda-setting theory	Valid

### 7.1 Findings

The current results can be considered from the perspective of the first three stages of the information lifecycle, as follows: (1) In accord with lifecycle theory, the dissemination of COVID-19-related news was a dynamic process, with different characteristics during the budding stage, spread stage, and outbreak stage. In the budding stage of COVID-19-related news, the number of reports about unknown pneumonia was small, and these failed to attract the attention of the media and the public. (2) When news reports emerged indicating that the cause of the unknown pneumonia was a novel coronavirus, the spread stage of the information lifecycle began, and when people.cn reported that “the novel coronavirus pneumonia can be transmitted from person to person and can infect doctors,” public opinion rapidly spread and COVID-19-related news entered the outbreak stage. (3) In a short period of time, the number of news reports grew rapidly, and the high degree of attention led to an increase in network energy and influence area. Opinion leaders, led by official media outlets, broadcasted the epidemic situation in a timely manner and refuted self-published media rumors in real time to avoid confusing the public with false information and causing panic.

Time-series visualization analysis revealed the following findings: (1) Keywords such as “Expert group,” “Wuhan,” and “Infection” appeared the earliest, and “Cure,” “Recovery,” and “Leave hospital” appeared the latest. (2) The rapid spread of COVID-19-related news in a short period of time led to a proliferation of public opinion expressed online. The subjects of public opinion and public focus changed over time, and even reversed in some cases. (3) Combined with agenda-setting theory, as the main opinion leaders, the official media play an important role in the spread of epidemic information and bear the responsibility of monitoring public opinion and persuasion after an event.

Social network analysis yielded the following observations: (1) The social network of COVID-19-related news was generally in a complete state because the network information volume and scale were large, and weak connections between the network edge and the core diluted the network density. (2) The strongest centrality of nodes such as “COVID-19,” “Pneumonia,” and “Viral infection” indicates that these nodes were at the core of the network. As a bridge between the dissemination of news and public opinion, the core words in the network are closely connected and can interact efficiently with other nodes. (3) Structural holes analysis indicated that the nodes exhibited non-redundant connections. The minimum constraint degree of “COVID-19” indicated that nodes exhibited the strongest control over the network via structural holes, whereas the constraint degree of “Pneumonia,” “Sick cases,” “Viral infection,” and other nodes increased in turn.

Overall, using lifecycle theory, we explored the different characteristics of COVID-19-related headlines at the budding, spread, and outbreak stages, as well as the impact on society at different stages. We dissected the role of mainstream media during the outbreak of epidemic-related public opinion, and found that mainstream media, as opinion leaders, can guide public opinion in a positive and active direction. Drawing on agenda-setting theory, we examined how objective reporting of the latest developments in the epidemic by Internet-based media helped to ease public tension and dispel people’s fears in the face of the sudden outbreak of COVID-19. Officials informed the public about the latest developments of the epidemic as needed, and addressed the concerns of the public in a timely manner, to maintain the stable operation of society.

#### 7.2 Limitations

The present study involved several limitations that should be considered. First, because we only analyzed the first wave of COVID-19-related news, the sample in this study was small and the cycle was incomplete. Second, the development of COVID-19 has been unpredictable, and it is difficult for the media to take into account all influencing factors regarding public opinion in their reports. In a follow-up study, we plan to analyze COVID-19 epidemic-related news over the complete lifecycle to further improve the accuracy of the conclusions.

## Supporting information

S1 File The relevant data in the manuscript. (ZIP)
