# Supplementary material for: The impact and management of internet-based public opinion dissemination during emergencies: A case study of Baidu News during the first wave of coronavirus disease 2019 (COVID-19)
Source: PLoS One. 2024 Apr 4;19(4):e0299374. doi: 10.1371/journal.pone.0299374 (PMC10994342; doi:10.1371/journal.pone.0299374)
Supplement: S1 File — (ZIP) [file pone.0299374.s001.zip › S1-Supporting information PONE-D-23-09860R1/URL-The Baidu News internet headlines of COVID-19.docx]

**1. The Baidu News internet headlines of COVID-19 are online available at:**

<https://www.baidu.com/s?rtt=1&bsst=1&cl=2&tn=news&word=%E6%96%B0%E5%9E%8B%E5%86%A0%E7%8A%B6%E7%97%85%E6%AF%92>

**2. Data availability statement**

The data sets generated and analyzed during the current study are available via the protocols.io ( <https://protocols.io/view/plosone-c5ify4bn> ),

github ( <https://github.com/ws8228/BaiduNews.git> ),

and are available from the corresponding author on reasonable request.
